# Hypnotics and the Occurrence of Bone Fractures in Hospitalized Dementia Patients: A Matched Case-Control Study Using a National Inpatient Database

**DOI:** 10.1371/journal.pone.0129366

**Published:** 2015-06-10

**Authors:** Hiroyuki Tamiya, Hideo Yasunaga, Hiroki Matusi, Kiyohide Fushimi, Sumito Ogawa, Masahiro Akishita

**Affiliations:** 1 Department of Geriatric Medicine, Graduate School of Medicine, The University of Tokyo, Tokyo, Japan; 2 Department of Clinical Epidemiology and Health Economics, School of Public Health, The University of Tokyo, Tokyo, Japan; 3 Department of Health Policy and Informatics, Tokyo Medical and Dental University Graduate School of Medicine, Tokyo, Japan; Klinikum rechts der Isar - Technical University Munich - TUM, GERMANY

## Abstract

**Background:**

Preventing falls and bone fractures in hospital care is an important issue in geriatric medicine. Use of hypnotics is a potential risk factor for falls and bone fractures in older patients. However, data are lacking on the association between use of hypnotics and the occurrence of bone fracture.

**Methods:**

We used a national inpatient database including 1,057 hospitals in Japan and included dementia patients aged 50 years or older who were hospitalized during a period of 12 months between April 2012 and March 2013. The primary outcome was the occurrence of bone fracture during hospitalization. Use of hypnotics was compared between patients with and without bone fracture in this matched case-control study.

**Results:**

Of 140,494 patients, 830 patients suffered from in-hospital fracture. A 1:4 matching with age, sex and hospital created 817 cases with fracture and 3,158 matched patients without fracture. With adjustment for the Charlson comorbidity index, emergent admission, activities of daily living, and scores for level walking, a higher occurrence of fractures were seen with short-acting benzodiazepine hypnotics (odds ratio, 1.43; 95% confidence interval, 1.19–1.73; *P*<0.001), ultrashort-acting non-benzodiazepine hypnotics (1.66; 1.37–2.01; *P*<0.001), hydroxyzine (1.45; 1.15–1.82, *P*=0.001), risperidone and perospirone (1.37; 1.08–1.73; *P*=0.010). Other drug groups were not significantly associated with the occurrence of in-hospital fracture.

**Conclusions:**

Short-acting benzodiazepine hypnotics and ultrashort-acting non-benzodiazepine hypnotics may increase risk of bone fracture in hospitalized dementia patients.

## Introduction

Bone fracture following falls in hospitalized patients is an unresolved problem in geriatric medical management. Older patients, especially dementia patients, have higher risks of delirium, insomnia and day—night reversal with environmental changes related to hospital admission. Hypnotics and psychoactives are sometimes used to relieve these symptoms.

Studies have examined the risk of hypnotic drug use on fall and fracture. In a meta-analysis of 22 studies, fall was significantly associated with use of sedatives and hypnotics, neuroleptics and antipsychotics, antidepressants, and benzodiazepines, but was not associated with narcotics [1]. A retrospective analysis of 3683 patients demonstrated that fall was associated with use of hypnotics [2]. Several studies showed that zolpidem was significantly associated with a higher risk of fall and fracture [3, 4]. Another study showed a hip fracture risk associated with non-benzodiazepine hypnotics [5]. These previous studies were mostly performed in nursing homes or in the community in older patients. Falls in the acute care hospital setting occur at a higher rate than falls in nursing home settings [6–9]. Moreover, hospital falls are also most frequent in safety incident reports, and sometimes lead to negligence suits [10]. However, to our knowledge, there has been no study that simultaneously assessed various types of hypnotics and their risk of in-hospital fracture in a nationwide clinical setting. In this study, we performed a matched case-control study to analyze the association between use of various types of hypnotics and the occurrence of bone fracture in hospitalized dementia patients, using a national inpatient database in Japan. We focused on dementia for several reasons. First, older patients with dementia have a fall risk twice or more than that of older patients without dementia [11, 12]. Second, older patients with dementia also have three to eight times more injuries with falls and more often have a bad prognosis [13–15]. Third, wandering, as well as other behavioral and psychological symptoms of dementia, along with hypnotic and psychoactive agents used to relieve these symptoms, increases fall risks [16, 17]. The medications tend to become the problem in acute care hospitals in trying to prevent falls in dementia patients.

## Methods

### Setting and Participants

For this study, we used the Diagnosis Procedure Combination database. The details of the database have been described elsewhere [18–20]. Briefly, the database includes administrative claims data and discharge abstract data, collected from about 1000 participating hospitals across Japan. The database includes the following information: patient age and sex; main diagnoses, comorbidities at admission and complications after admission recorded according to the International Classification of Diseases, 10th Revision (ICD-10) and text data in Japanese; medical procedures; medicines and devices used; length of stay; activities of daily life (ADL) scores at admission; and discharge status. A unique identifier was used for each hospital. All patient identifiers were removed from this database. Because of the anonymous nature of the data, the Institutional Review Board of The University of Tokyo waived the need for written informed consent from the participants. Study approval was also obtained from the Institutional Review Board of The University of Tokyo.

Among approximately 7 million inpatients over 12 months between April 1, 2012 and March 31 2013, we identified patients aged 50 years or older. Of these, we selected patients who were diagnosed with dementia or dementia-related diseases, including dementia in Alzheimer disease (ICD-10 code, F00), vascular dementia (F01), dementia in Pick disease (F02.0, G31.0), dementia in Creutzfeldt—Jakob disease (F02.1, G810), dementia in Huntington disease (F02.2, G10), dementia in Parkinson disease (F02.3, G20), dementia in human immunodeficiency virus disease (F02.4, B220), dementia in other specified diseases classified elsewhere (F02.8), unspecified dementia (F03), alcoholic dementia (F107), dementia in cerebral lipidosis (F028, E756), Lewy bodies dementia (F028, G318), and mild cognitive disorder (F06.7).

We extracted data on the following 17 types of hypnotics (or sedatives used as hypnotics) for each patient: benzodiazepine anxiolytics; diazepam; ultrashort-acting benzodiazepine hypnotics; short-acting benzodiazepine hypnotics; middle- to long-acting benzodiazepine hypnotics; ultrashort-acting non-benzodiazepine hypnotics; melatonin-receptor agonists; hydroxyzine; phenothiazine antipsychotics; haloperidol; sulpiride; risperidone and perospirone; multi-acting-receptor-targeted antipsychotics used as a hypnotic; antidepressants used as a hypnotic; Japanese *kampo* herbal medicine used as a hypnotic and in the treatment of behavioral and psychological symptoms of dementia; and other neurological drugs used as hypnotics.

Comorbidities were assessed by ICD-10 codes and converted into scores to calculate the Charlson comorbidity index (CCI) based on Quan’s algorithm [21]. ADL scores for walking on a flat floor were also extracted including bedridden (Score 0), totally assisted (Score 1), partially assisted (Score 2) and without disability (Score 3).

### Outcomes

The outcome in this study was in-hospital fracture. In the database, comorbidities already present at admission are clearly differentiated from complications that occurred after admission. In-hospital fracture was defined as fracture that occurred after admission and was determined according to the following ICD-10 codes: fracture of skull and facial bones (S02); fracture of neck (S12); fracture of rib(s), sternum and thoracic spine (S22); fracture of lumbar spine and pelvis (S32); fracture of shoulder and upper arm (S42); fracture of forearm (S52); fracture at wrist and hand level (S62); fracture of femur (S72); fracture of lower leg, including ankle (S82); fracture of foot, except ankle (S92); fractures involving multiple body regions (T02); fracture of spine, level unspecified (T08); fracture of upper limb, level unspecified (T10); and fracture of lower limb, level unspecified (T12).

### Statistical Analyses

We performed a matched case—control study. First, we identified cases with in-hospital fracture. For each case, we selected four controls of similar age (±5 years) and the same sex from the same hospital. When there were more than four matched-control candidates to each case, we randomly selected four control patients. Specifically, control cases were sorted by randomly generated values from a Microsoft SQL server and the top four were selected. There are two ways to conduct matching: matching with replacement; and matching without replacement [22]. Matching with replacement means that controls can be used as matches for more than one treated individual; matching without replacement signifies that controls cannot be used as matches for more than one treated individual. Though the statistical analysis becomes more complex, matching with replacement can often decrease bias because controls that resemble many treated individuals can be used multiple times [23, 24]. Moreover, the order of matching the treated individuals is immaterial in the case of matching with replacement. One methodological paper compared matching with and without replacement in three matching methods, and it found that matching with replacement had a smaller bias among all three methods [25]. Thus, we chose matching with replacement for the present study. If a control case was a candidate for more than one case, we included both matches. In the following analysis, one control was selected three times, 95 controls were selected twice, and they were weighted using frequency weights. If the number of matched-control candidates for each case was less than four, we also included both the corresponding case (62 cases) and control (138 controls) in the analytical group subset to avoid selection bias, unless no control subjects were assigned (13 cases).

Descriptive statistics were presented for the matched patients. Categorical variables were compared using the chi square test. We performed multivariable logistic regression for the occurrence of in-hospital fractures fitted with a generalized estimating equation to account for the clustered nature of the cases and controls. There are two ways to cluster in matched case-control studies: generalized estimating equations (GEEs) and conditional logistic analysis. Both methods can make consistent estimates. As GEE is more robust in terms of the specification of matching effect, we chose GEEs [26]. The dependent variable was in-hospital fracture, and independent variables included, emergent admission, ADL score for walking on a flat floor, CCI and 17 classes of drugs. All statistical analyses were conducted using IBM SPSS version 22.0 (IBM SPSS, Armonk, NY, USA).

## Results

Among 140,494 eligible patients, 830 patients suffered from in-hospital fracture.

Using 1:4 matching, we obtained a case group of 817 patients and a control group of 3158 patients. Table 1 shows the baseline characteristics of the matched patients (n = 3975). As a result of matching, there was no significant difference in age (*P* = 0.582) or sex (*P* = 0.728) between the case and control groups. To exclude the possibility that controls may have been matched to cases with a larger age difference than to cases with a smaller age difference, we also compared the distribution of age in the case and control groups. Mean, median, standard deviation, range and interquartile range of age (years) in the case and control groups were 81.5 vs. 81.8, 82.0 vs. 82.0, 7.9 vs. 7.5, 50–103 vs. 50–103, 11.0 vs. 10.0, respectively. The age distributions of the case and control groups were also similar. No significant difference in CCI or emergent admission was present between the cases and controls. The ADL score for walking on a flat floor on admission was significantly different between the groups.

**Table 1:** Characteristics of patients in the matched case and control groups.

		Cases (n = 817)		Controls (n = 3158)		*P*
Charlson comorbidity index	0	141	17.3%	526	16.7%	0.971
	1	264	32.3%	1013	32.1%	
	2	199	24.4%	805	25.5%	
	3	115	14.1%	435	13.8%	
	≥4	98	12.0%	379	12.0%	
Emergent admission		415	50.8%	1538	48.7%	0.286
ADL score (walking on flat floor)	0 (bedridden)	459	56.3%	1773	56.3%	0.001
	1 (totally assisted)	59	7.2%	208	6.6%	
	2 (partially assisted)	105	12.9%	382	12.1%	
	3 (without disability)	113	13.9%	588	18.7%	
	Unknown	81	9.9%	207	6.5%	

Table 2 shows 17 types of hypnotics (or sedatives used as hypnotics) used for the case and the control groups. The most frequently used drugs in both groups were ultrashort-acting non-benzodiazepine hypnotics, followed by short-acting benzodiazepine hypnotics. The proportion of patients who used any of the 17 types of drugs was significantly higher in the case than the control group (66.8% vs. 51.9%, *P*<0.001) (Table 2). The proportion of patients who used more than three types of hypnotics (or sedatives used as hypnotics) was also higher in the case than the control group (24.2% vs. 14.6%, *P*<0.001). The case group was significantly more likely to use benzodiazepine anxiolytics; ultrashort-acting benzodiazepine hypnotics; short-acting benzodiazepine hypnotics; middle- to long-acting benzodiazepine hypnotics; ultrashort-acting non-benzodiazepine hypnotics; melatonin-receptor agonists; hydroxyzine; phenothiazine antipsychotic; haloperidol; sulpiride; risperidone and perospirone; multi-acting-receptor-targeted antipsychotics used as a hypnotic; and an antidepressant used as a hypnotic. The proportion of patients who used Japanese *kampo* herbal medicine was not significantly different between the cases and controls.

**Table 2:** Comparison of drug use between matched case and control groups.

	Case (n = 817)		Control (n = 3158)		*P*
benzodiazepine anxiolytic	98	12.0%	279	8.8%	0.006
diazepam	46	5.6%	171	5.4%	0.809
ultrashort-acting benzodiazepine hypnotic	24	2.9%	55	1.7%	0.029
short-acting benzodiazepine hypnotic	173	21.2%	460	14.6%	<0.001
middle- to long-acting benzodiazepine hypnotic	57	7.0%	159	5.0%	0.029
ultrashort-acting non-benzodiazepine hypnotic	194	23.7%	461	14.6%	<0.001
melatonin-receptor agonist	34	4.2%	83	2.6%	0.021
hydroxyzine	119	14.6%	288	9.1%	<0.001
phenothiazine antipsychotic	25	3.1%	58	1.8%	0.029
haloperidol	124	15.2%	314	9.9%	<0.001
sulpiride	26	3.2%	54	1.7%	0.008
risperidone and perospirone	144	17.6%	343	10.9%	<0.001
multi-acting-receptor-targeted antipsychotics used as hypnotic	77	9.4%	217	6.9%	0.013
antidepressant used as hypnotic	50	6.1%	103	3.3%	<0.001
Japanese *kampo* herbal medicine used as hypnotic and in treatment of BPSD	50	6.1%	192	6.1%	0.966
other neurological drugs used as hypnotic	31	3.8%	91	2.9%	0.178
Number of drugs					
0	271	33.2%	1519	48.1%	<0.001
1	201	24.6%	746	23.6%	
2	147	18.0%	433	13.7%	
≥3	198	24.2%	460	14.6%	

Table 3 shows the results of the multivariable logistic regression analysis. A higher occurrence of in-hospital fracture was significantly associated with use of a short-acting benzodiazepine hypnotic, an ultrashort-acting non-benzodiazepine hypnotic, a hydroxyzine, risperidone and perospirone. Neither melatonin agonists nor Japanese *kampo* herbal medicine was associated with the occurrence of in-hospital fracture.

**Table 3:** Generalized estimating equation analysis result.

	odds ratio	95% confidence interval			P
Type of admission					
Non-emergent	Reference				
Emergent	1.03	0.88	–	1.20	0.712
ADL score of walking on flat floor					
0 (bedridden)	Reference				
1 (totally assisted)	1.01	0.75	–	1.37	0.924
2 (partially assisted)	1.00	0.79	–	1.27	0.989
3 (without disability)	0.67	0.54	–	0.85	0.001
Unknown	1.42	1.08	–	1.86	0.011
Charlson comorbidity index					
0	Reference				
1	0.91	0.72	–	1.16	0.461
2	0.88	0.69	–	1.12	0.296
3	0.93	0.71	–	1.22	0.609
≥4	0.91	0.68	–	1.22	0.526
benzodiazepine anxiolytic	1.15	0.90	–	1.47	0.250
diazepam	0.91	0.65	–	1.29	0.608
ultrashort-acting benzodiazepine hypnotic	1.53	0.94	–	2.50	0.086
short-acting benzodiazepine hypnotic	1.43	1.19	–	1.73	0.000
middle- to long-acting benzodiazepine hypnotic	1.01	0.70	–	1.45	0.977
ultrashort-acting non-benzodiazepine hypnotic	1.66	1.37	–	2.01	0.000
melatonin-receptor agonist	1.25	0.84	–	1.88	0.273
hydroxyzine	1.45	1.15	–	1.82	0.001
phenothiazine antipsychotic	1.06	0.57	–	2.00	0.847
haloperidol	1.16	0.91	–	1.48	0.244
sulpiride	1.57	0.95	–	2.57	0.077
risperidone and perospirone	1.36	1.08	–	1.73	0.010
multi-acting-receptor-targeted antipsychotics used as hypnotic	1.07	0.79	–	1.44	0.654
antidepressant used as hypnotic	1.38	0.97	–	1.98	0.077
Japanese *kampo* herbal medicine	0.72	0.52	–	1.00	0.052
other neurological drugs used as hypnotic	1.10	0.62	–	1.96	0.736

## Discussion

The present study showed an increased risk for in-hospital fracture with several hypnotics and psychoactives used as hypnotics in dementia patients who were admitted to acute care hospitals. In-hospital fracture risk was associated with a short-acting benzodiazepine hypnotic, an ultrashort-acting non-benzodiazepine hypnotic, and risperidone and perospirone.

A previous US study of nursing home residents demonstrated a fracture risk with ultrashort-acting non-benzodiazepine hypnotics [5]. The present study also showed a risk with ultrashort-acting non-benzodiazepine hypnotics. A new finding in the present study is that shorter-acting drugs had relatively higher odds ratios for in-hospital fracture than longer-acting drugs. A possible explanation for this may be that patients are more likely to fall when drowsy soon after taking hypnotics. Another possible reason is that physicians may have avoided prescribing long-acting hypnotics for frail patients.

To our knowledge, the present study is the first to show that hydroxyzine may increase the risk of fracture in dementia patients. Hydroxyzine has been shown to be as effective as bromazepam, one of the benzodiazepines, in the treatment of generalized anxiety disorder [27]. Because hydroxyzine has a half-life of around 3 hours, it may act like a short-acting benzodiazepine.

Our results showed that a melatonin agonist was not significantly associated with the occurrence of in-hospital fracture. Melatonin-receptor agonists including ramelteon are new types of hypnotics. They act on the GABA_A_ receptor-independent pathway in contrast to most of hypnotics that act on the GABA_A_ receptor. The present study suggests that a melatonin agonist may be safer than other hypnotics in terms of fall and fracture risk.

Japanese *kampo* herbal medicines, which are often used as sedatives or hypnotics in Japan, have beneficial effects on the behavioral and psychological symptoms of dementia [28–30]. Our results suggest that these drugs would be good alternatives to conventional hypnotics or sedatives in dementia patients and may reduce fracture risk.

This study has several limitations. First, recorded diagnoses in an administrative claims database are less well validated than those in planned prospective studies. Second, the time interval between drug administration and related in-hospital fracture cannot be identified from the database and its causal relationship remains to be clarified. We have information on the timing and use of these agents, but not on the timing of fracture. Consequently, we are not certain as to whether the agent was prescribed before or after the fracture, or if it was not used for a short period of time for weeks to months prior to fracture. Third, it is difficult to distinguish the deleterious effect of hypnotic use itself from underlying conditions, including night delirium and insomnia requiring prescription of hypnotics. Fourth, there was no information about previous falls, and so we were unable to examine this relationship owing to the lack of data.

In light of these findings, it is preferable to avoid prescribing short-acting benzodiazepines and ultrashort-acting non-benzodiazepine hypnotics, risperidone or perospirone, hydroxyzine, or multi-acting-receptor-targeted antipsychotics to in-hospital dementia patients. Melatonin-receptor agonists or Japanese *kampo* herbal medicine may be preferable to these drugs.

## Conclusion

Short-acting benzodiazepines and ultrashort-acting non-benzodiazepine hypnotics were associated with an increase in in-hospital fractures in dementia patients, while no significant association with an increase in in-hospital fractures was seen with middle- to long-acting benzodiazepine hypnotics, melatonin-receptor agonists, or Japanese *kampo* herbal medicine.
